# Neural Network Models for Bitcoin Option Pricing

**DOI:** 10.3389/frai.2019.00005

**Published:** 2019-07-03

**Authors:** Paolo Pagnottoni

**Affiliations:** Department of Economics and Management, University of Pavia, Pavia, Italy

**Keywords:** cryptocurrencies, bitcoin, option pricing, neural network, alternative option pricing methods

## Abstract

Despite the current growing interest in Bitcoins—and cryptocurrencies in general—financial instruments, as well as studies related to them, are quite underdeveloped. Therefore, this article aims to provide a suitable pricing model for options written on this peculiar underlying. This is done through an artificial neural network approach, where classical pricing models—namely the trinomial tree, Monte Carlo simulation, and explicit finite difference method—are used as input layers. Results show that options written on Bitcoin turn out to be systematically overpriced when considering classical methods, whereas a noticeable improvement in price predictions is achieved by means of the proposed neural network model.

## 1. Introduction

Stock options are a category of financial derivatives which became widely employed by investors and speculators during the last few decades. Nevertheless, investors may ineffectively manage their portfolios if they are not able to value options in a proper way. For this reason, a reliable methodology capable to yield an option's current price or forecast is fundamental for investors in order to produce a rigorous decision making. This is particularly true when considering non-mature and volatile markets like the cryptocurrency one.

The theory of option pricing is broad and involves various types of pricing techniques, largely parametric ones. The most widely known option pricing method is arguably the one defined by Black and Scholes (1973). Although this technique has been widely employed by practitioners, its strict set of assumptions, as well as subjectivity with respect to the parameter choices, often yields to unreliable results to some extent. To illustrate, the leptokurtic behavior of return distributions and the volatility smiles and skews are features that cannot be captured by such a simplistic technique.

Besides the Black-Scholes model and its modifications, other parametric models have been developed and became widely used, among which the (binomial and trinomial) tree models, the finite difference method and the Monte Carlo simulation. While tree models converge to the Black-Scholes one in case the time occurring between steps is small enough, other methodologies take into consideration pricing aspects that these two models do not. Indeed, the Monte Carlo simulation allows for random shocks other than those provided by the volatility and the movement probabilities of the tree models, whereas the finite difference method relies on a different simulation scheme. This is the reason why in this paper examines and includes tree models, the Monte Carlo simulation, and the finite difference method as pricing methodologies.

Alongside the category of classical derivative and option pricing models, non-parametric models, such as neural networks gradually emerged, mainly thanks to their improved predictive performance with respect to the former techniques. Yao et al. (2000) predicts prices related to the Nikkei 225 index futures using back-propagation neural networks. Their results show that, despite the Black-Scholes model is still good for pricing at-the-money options, the neural network outperforms it, in particular when considering volatile markets. Another research conducted by Liang et al. (2009) motivates this paper's approach, as the authors use classical models (binomial tree, finite difference method, and Monte Carlo simulation) in a first stage to forecast the option price and refine those forecasts through neural networks and support vector machines in a second stage. This technique allows to notably reduce forecast error, i.e., substantially improves price forecasts in their Hong Kong option market framework. Nonetheless, there are many other examples on neural network models for derivative securities pricing which found that neural networks outperform classical models—see, for instance, Hutchinson et al. (1994), Malliaris and Salchenberger (1996), Amilon (2003), Binner et al. (2005), and Lin and Yeh (2005).

Research related to the cryptocurrency market, as the phenomenon itself, is relatively new. Despite that, there is a massive interest of the academic community in investigating this new market and its peculiar features from all points of view, with a particular focus on Bitcoin. Indeed, since Nakamoto (2008) introduced the concept of Bitcoin as a purely peer-to-peer version of electronic cash, researches developed following different and multidisciplinary fields. Some researchers provide a general descriptional analysis of the cryptocurrency framework. To illustrate, in Dwyer (2015) we may find a detailed overview on technical issues of Bitcoin and the cryptocurrency market in general. Also White (2015) goes through the key concepts of cryptocurrencies, while focusing on the so called “Altcoins”^1^. A further study by Kroll et al. (2013) examines the Bitcoin mining process thoroughly. Another stream of the literature, with studies conducted by Brandvold et al. (2015) and Pagnottoni and Dimpfl (2019), finds the leader and follower Bitcoin exchanges of the price discovery process through an econometric analysis of its price across different exchange.

Despite the quite wide set of studies in the cryptocurrency area, to the best of our knowledge there is not yet any research trying to address option pricing related to Bitcoin (or cryptocurrency) derivatives. The aim of this study is to propose a pricing methodology that is feasible to price cryptocurrency options. Without loss of generality, the paper focuses on european style Bitcoin put and call options which became recently available on the market. To this end, the study makes use of a two stage approach. The first stage consists of option pricing through parametric approaches, such as tree models, finite difference method, and Monte Carlo simulation. In the second stage, artificial neural networks are employed in order to combine the parametric option pricing approaches and capture the residual errors by learning schemes in the current status of the option market. Their performance is then compared to the conventional option pricing techniques obtained in the first stage. Results point to the predominance of the neural network models with respect to the conventional methods in pricing Bitcoin options and, therefore, in capturing their real price dynamics. As a robustness check, an out-of-sample analysis confirm the previous result, as well as a cross validation analysis through random sub-sampling reveals that—despite there is still some room for improvement—results are arguably stable and the neural network is a suitable model in order to price options written on Bitcoin.

The remainder of the paper proceeds as follows. Section 2 outlines the methodology employed. Section 3 describes and analyzes the data. Section 4 presents the results. Section 5 illustrates the robustness analysis conducted. Section 6 concludes.

## 2. Methodology

This section briefly introduces the classical parametric option pricing techniques used in this paper: specifically, tree models, finite difference method, and Monte Carlo simulation. After that, I discuss the neural network model and the comprehensive approach for option pricing.

The following notation will be used. *S* represents the underlying asset price, *C* is the option price, *K* is the options' exercise price, σ denotes the asset price volatility, *r* represents the risk-free interest rate, Δ*t* is the time interval (i.e., the time period length), and *T* is the time to maturity.

### 2.1. Tree Models

Tree models are widely used not only to price European style options, but also closed-form American options, as they can account for the early exercise feature. Milestone references for binomial trees are the ones of Cox et al. (1979) and Rendleman and Bartter (1979). Further extensions are proposed by Boyle (1977), Nelson and Ramaswamy (1990), and Hull and White (1990a).

In the binomial tree setup, the underlying asset price *S*_*t, i*_ with *t* = 0, 1, 2, …, *n*−1 may either experience an up movement to *S*_*t*+1, *i*_ or a down movement to *S*_*t*+1, *i*+1_, with *t* = 1, 2, …, *n*. This happens according to an upward rate *u* and a downward rate *d*, which Cox et al. (1979) define as:

(1) $$\begin{array}{l} u=e^{\sigma \sqrt{△t}},\;d=e^{-\sigma \sqrt{△t}} \end{array}$$

where $△t=\frac{T}{n}$ denotes the time step from *t* to *t* + 1 and *n* the total number of time steps in the binomial tree.

**Figure 1:**
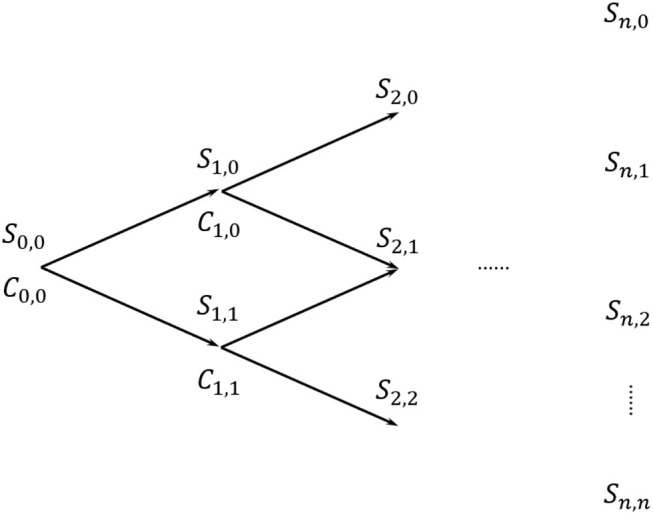
Binomial tree.

A graphical representation of a *n*-step binomial tree is illustrated in Figure 1. Arrows constitute possible paths for the price dynamics, whereas nodes represent the underlying price *S*_*t, i*_ from which the option price *C*_*t, i*_ is computed. Option prices are then recursively computed from the last ones to the first one, going backwards, according to the following:

(2) $$\begin{array}{l} C_{t-△t,i}=e^{-r△t}\left(pC_{t,i+1}+\left(1-p\right)C_{t,i}\right) \end{array}$$

where *r* is the risk-free rate, and the probabilities of up (*p*) and down (*p*_*d*_) movements are defined as

(3) $$\begin{array}{l} p=\frac{e^{r△t}-d}{u-d},\;p_{d}=1-p \end{array}$$

**Figure 2:**
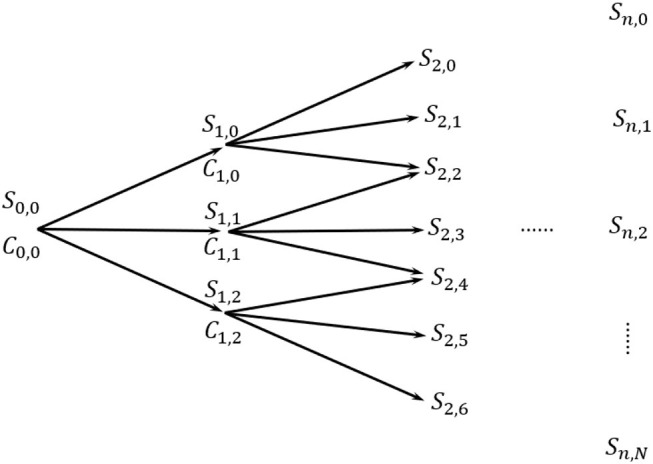
Trinomial tree.

The trinomial tree (Figure 2) works in a similar way. However, in this setup, the underlying asset price *S*_*t, i*_ with *t* = 0, 1, 2, …, *n* − 1 may either experience an up movement to *S*_*t*+1, *i*_, a middle movement to *S*_*t*+1, *i*+1_ or a down movement to *S*_*t*+1, *i*+2_, with *t* = 1, 2, …, *n*. This happens according to an upward rate *u*, downward rate *d* and middle rate *m* defined as:

(4) $$\begin{array}{l} u=e^{\sigma \sqrt{2△t}},\;d=e^{-\sigma \sqrt{2△t}},\;m=1 \end{array}$$

In this case, the probabilities of up (*p*), down (*p*_*d*_) and middle (*p*_*m*_) movements are defined as:

(5) $$\begin{array}{l} p={\left(\frac{e^{\left(r\right)\frac{\Delta t}{2}}-e^{-\sigma \sqrt{\frac{\Delta t}{2}}}}{e^{\sigma \sqrt{\frac{\Delta t}{2}}}-e^{-\sqrt{\frac{\Delta t}{2}}}}\right)}^{2},\;p_{d}={\left(\frac{e^{\sigma \frac{\Delta t}{2}}-e^{\left(r\right)\frac{\Delta t}{2}}}{e^{\sigma \sqrt{\frac{\Delta t}{2}}}-e^{-\sqrt{\frac{\Delta t}{2}}}}\right)}^{2},\\ p_{m}=1-\left(p+p_{d}\right) \end{array}$$

Among the advantages of using the trinomial trees, computational efficiency as well as precision are of our interest. Indeed, the trinomial tree should yield to more precise prices with less time steps if compared to the binomial counterpart.

### 2.2. Finite Difference Method

As extensively described in Brennan and Schwartz (1977), the finite difference method allows to price options through the solution of some differential equations with respect to the option prices. These equations are transformed into difference equations, whose solutions are iteratively solved by CPUs.

According to the finite difference method, the time to maturity *T* is segmented into *p* equally sized time periods Δ*t*, whereas the asset price is segmented into *q* steps of length Δ*S*, ranging from a minimum of 0 to a maximum of *S*_*max*_. This can be represented as a grid in which the horizontal line is the number of periods and the vertical one the asset prices.

In the present case, the application uses the so called explicit finite difference method, which solves the differential equations in a forward way, as elucidated by Hull and White (1990b). The reason behind our choice is that the explicit finite difference method is arguably more efficient than the implicit one, which in contrast solves the differential equations backwards. In particular, the equation to be solved is the well-known partial differential equation of Black-Scholes, i.e.,

(6) $$\begin{array}{l} \frac{\partial C}{\partial t}+\frac{1}{2}\sigma^{2}S^{2}\frac{\partial^{2}C}{\partial S^{2}}+rS\frac{\partial C}{\partial S}=rC \end{array}$$

Where *i* = 1, 2, …, *p* and *j* = 1, 2, …, *q*. The discrete version of Equation (6) is:

(7) $$\begin{array}{l} \begin{array}{c} -\frac{C_{i,j}-C_{i-1,j}}{\Delta t}=\frac{1}{2}\sigma^{2}\frac{C_{i,j+1}-2C_{i,j}+C_{i,j-1}}{\Delta S^{2}}+\\ \;+rS\frac{C_{i,j}-2C_{i,j-1}}{2\Delta S}-rC_{i+1,j}. \end{array} \end{array}$$

The option price can then be derived as:

(8) $$\begin{array}{l} C_{i,j}=\frac{1}{1+r\Delta t}\left(pC_{i+1,j+1}+p_{m}C_{i+1,j}+p_{d}C_{i+1,j-1}\right) \end{array}$$

where the probabilities associated with an up, middle or down movement are respectively:

(9) $$\begin{array}{l} p=S_{j}r\frac{\Delta t}{2\Delta S}+\frac{1}{2}S_{j}^{2}\sigma^{2}\frac{\Delta t}{\Delta S^{2}} \end{array}$$

(10) $$\begin{array}{l} p_{m}=1-S_{j}^{2}\sigma^{2}\frac{\Delta t}{\Delta S^{2}} \end{array}$$

(11) $$\begin{array}{l} p_{d}=-\frac{S_{j}r\Delta t}{2\Delta S}+\frac{1}{2}S_{j}^{2}\sigma^{2}\frac{\Delta t}{\Delta S^{2}} \end{array}$$

For a detailed explanation of the finite difference method, refer to Brennan and Schwartz (1977) and Hull and White (1990b).

### 2.3. Monte Carlo Simulation

The Monte Carlo simulation is used to obtain the underlying asset price at the option maturity by means of averaging a sufficiently high number of stochastic asset price paths, obtained by assuming that the underlying price follows a log-normal distribution, that is simulating *L* scenarios for the underlying price evolution as:

(12) $$\begin{array}{l} S_{T}=S_{t}e^{\left(r-\frac{1}{2}\sigma \right)\left(T-t\right)+\sigma \sqrt{T-t}\Delta W_{t}} \end{array}$$

where *W*_*t*_ denotes a standard Wiener process at time *t*.

After that, option prices are found by discounting that average result backwards. In other words, given the payoffs at maturity *T* of call and put options, respectively as:

(13) $$\begin{array}{l} C_{T}=max\left(0,S_{T}-K\right),\;P_{T}=max\left(0,K-S_{T}\right) \end{array}$$

the resulting call and put prices are obtained as an average of the *L* simulated scenarios, i.e.,

(14) $$\begin{array}{l} C_{t}=\frac{1}{L}\overset{L}{\underset{l=1}{\sum }}C_{l},\;P_{t}=\frac{1}{L}\overset{L}{\underset{l=1}{\sum }}P_{l} \end{array}$$

where *l* = 1, 2, …, *L*.

### 2.4. Neural Networks to Improve Precision

Option prices dynamics depend on several variables as well as on an economic environment and rules that continuously change. Despite parametric methods mimic the behavior of real option prices, it may be argued that they do not fully reflect the actual market evolution of option prices.

**Figure 3:**
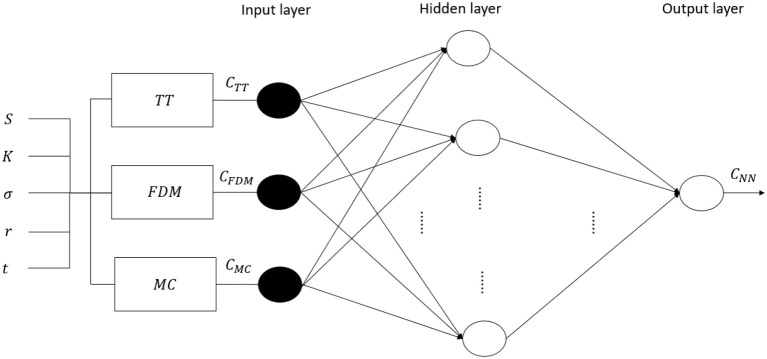
The multilayer perceptron neural network model. The following notation is used: NN stands for the neural network model, TT corresponds to the trinomial tree, FDM represents the finite difference method, and MC for the Monte Carlo simulation.

To cope with that, similarly to Liang et al. (2009), this paper defines a two-step procedure in order to consistently evaluate option prices. The first step consists of pricing options according to the three parametric methods described above, i.e., tree models, finite difference method, and Monte Carlo simulation. The prices obtained in the first step are then used as input training vector of a neural network model in the second step. As a consequence, once the main information regarding an option's price are captured through the parametric methods in the first step, the machine learning neural network can concentrate its modeling power to approximate the non-linear features of the option pricing errors. A graphical representation of the model can be found in Figure 3.

It is well-known that the option market is a complex system with non-linear characteristics. This further motivates our approach, since the use of a particular kind of neural network model, the multilayer perceptron one, allows to account for these features. Indeed, through the multilayer perceptron neural network one is able to include include hidden layers and non-linear activation functions that may capture the non-linearity of the option market. An organic description of multilayer perceptron neural networks can be found, for example, in Haykin et al. (2009).

### 2.5. Performance Assessment

In this subsection the the assessment criteria used to evaluate our models are presented. Performances of our pricing methods are judged according to three widely employed measures, i.e., the mean absolute error (MAE), mean squared error (MSE), and the mean absolute percentage error (MAPE). These criteria are defined by

(15) $$\begin{array}{l} MAE=\frac{1}{N}\overset{N}{\underset{n=1}{\sum }}|A_{t,n}-F_{t,n}| \end{array}$$

(16) $$\begin{array}{l} MAPE=\frac{1}{N}\overset{N}{\underset{n=1}{\sum }}|\frac{A_{t,n}-F_{t,n}}{A_{t,n}}| \end{array}$$

(17) $$\begin{array}{l} MSE=\frac{1}{N}\overset{N}{\underset{n=1}{\sum }}{\left(A_{t,n}-F_{t,n}\right)}^{2} \end{array}$$

where *A* is the actual option value and *F* is the fitted value obtained by the corresponding pricing model, being *t* the specific time at which the option is evaluated and *N* the number of observations.

## 3. Data

An option market for cryptocurrencies—and Bitcoin—is gradually emerging. I analyze data from deribit.com, a platform offering trading of futures and European style options written on Bitcoin. In particular, the corresponding underlying on which the options are written consists of the deribit BTC index^2^.

Data are collected from 16 May 2018 to 15 July 2018, on a daily basis, every day at the same time (11:00 UTC). To be precise, the retrieved data are the deribit BTC index and all available option prices related to that day (European calls and puts).

Following Liang et al. (2009) the analysis is restricted to options having a time to maturity comprised between 5 and 20 days, as well as to in-the-money options having a spread which is lower than 50%. In this way it is possible to overcome price fluctuations related to the expiration effect and liquidity problems linked to the long term time to maturity options, as well as to eliminate outliers reflecting expectations which are somehow not rational and may heavily affect results. Furthermore, the choice of such a maturity range is in line with the peculiar short term feature of cryptocurrency options, whose maturities are generally smaller than the ones related to traditional option markets. To illustrate, the majority of options in our full dataset were issued only 8 days before maturity.

Given the set of restrictions adopted above, the dataset ends up with a total number of 281 call and 695 put prices. In the current analysis, the first 10 weeks will be used for the estimation purposes, while the last 2 weeks will be used for out-of-sample performance assessment.

As far as the parameter specifications, a 15-days historical volatility for the deribit BTC index and the 2-months Libor interest rate as risk-free rate are used. Moreover, the finite difference method has a grid of size 3*T* and the Monte Carlo simulation involves 10,000 repetitions.

The neural network involves several specifications, too. Firstly, the study relies on the widely spread backpropagation algorithm for the parameter estimation. Secondly, the most widely employed activation functions are tested in order to choose the one ensuring the best performance in terms of fitting^3^. Results indicate that the sigmoid function is the one ensuring the smallest sizes of prediction error. Thirdly, an analysis of the optimal number of hidden layers and neurons in the network is conducted, following the iterative procedure described in Stathakis (2009). Results suggest a model having two neurons and one hidden layer.

## 4. Empirical Findings

### 4.1. Experimental Results on Selected Options

In this section results are presented distinguishing between call and put options.

**Figure 4:**
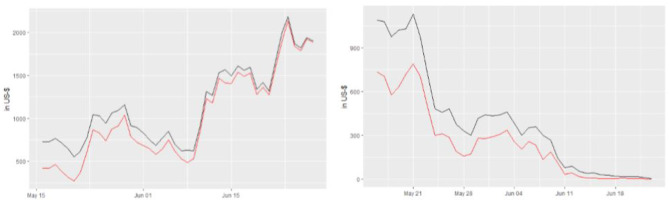
Real option prices (black) against trinomial tree price predictions (red) for the option expiring on 29 June 2018, *K* = 8000, call (left) and put (right).

Without loss of generality, a plot of a representative option price evolution against one of the parametric methods (the trinomial tree) prediction is shown in Figure 4. Overall, classical parametric option pricing methods (i.e., trinomial tree, finite difference method and Monte Carlo simulation) lead to price predictions which are consistently lower than the actual option prices, both in the put and the call cases. Consequently, it may be argued that options written on Bitcoin are systematically overpriced by the platform when considering the parametric methods in question. Notwithstanding this, theoretical prices yielded by parametric methods converge to the real option prices as the time to maturity becomes smaller. This is in line with the behavior of the traditional markets for option exchanges, where a small time to maturity leads to a convergence of theoretical and real option prices.

**Figure 5:**
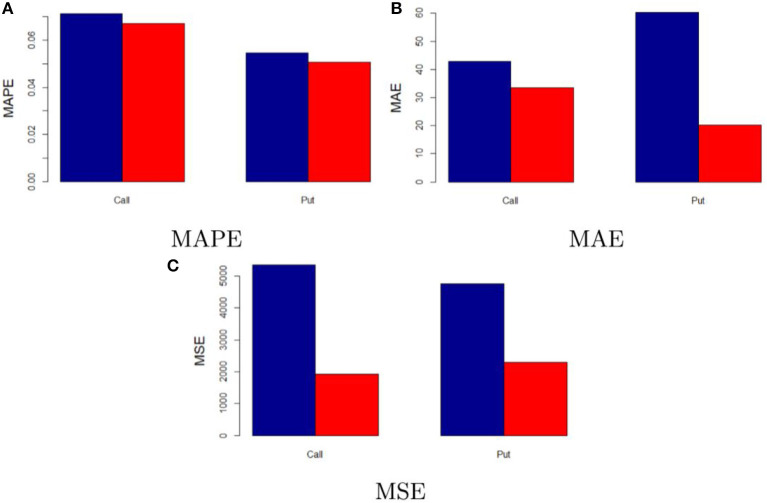
In-sample performance of neural network and “best” classical model. The figure compares the in-sample performance of the neural network model (red) and “best” classical model (blue). **(A–C)** Illustrate the MAPE, MAE, and MSE, respectively.

Prediction errors associated with each category of options are illustrated in Table 1. Absolute and relative model performance measures are quite comparable across the considered classical parametric methods. Besides that, it is clear that the neural network outperforms them in terms of prediction accuracy. This is also graphically represented in Figure 5, which shows the model performance metrics of the neural network against those of the “best” classical model, meaning the parametric model among the ones used in this study showing the lowest prediction error. To illustrate, when comparing the neural network and the “best” classical model performances the MAPE lowers by 6% in the call case and 7.33% in the put one, the MAE by 21.58% (call) and 0.4% (put) as well as the MSE by 64.07% (call) and 51.75% (put). This is mainly due to the fact that the multilayer perceptron neural network can deal with the complexity and non-linearity of the option market and the cryptocurrency market. Indeed, price predictions yielded in the first step by the conventional approach are then refined into the second step by the neural network, which focuses on lowering the errors existing between the real option prices and the predicted ones.

**Table 1:** In-sample performance of neural network and classical models.

**–**	**TT**	**FDM**	**MC**	**NN**
**CALL**				
MAPE	0.0713	0.0713	0.0716	0.0670
MAE	42.78	42.79	43.2	33.55
MSE	5,362.41	5,362.65	5,401.13	1,926.66
**PUT**				
MAPE	0.0546	0.0547	0.0546	0.0506
MAE	56.00	56.05	56.08	33.63
MSE	4,764.71	4,764.81	4,765.29	2,299.11

*The following notation is used: NN represents the neural network model, TT corresponds to the trinomial tree, FDM stands for finite difference method, and MC for the Monte Carlo simulation*.

The obtained results are in accord with the existing literature on option pricing through non-parametric methods and, particularly, neural networks—see Hutchinson et al. (1994), Malliaris and Salchenberger (1996), Amilon (2003), Binner et al. (2005), and Lin and Yeh (2005). Indeed, all these studies point to an overall predominance of neural network based models in pricing options with respect to conventional methodologies. It may be argued that this holds true also for particular markets like the cryptocurrency one, whose particular features are well-captured by non-parametric models, such as the neural network.

## 5. Robustness Analysis

With the aim of testing the robustness of our model, this section provides an out-of-sample performance analysis as well as a cross-validation analysis through repeated random sub-sampling.

### 5.1. Out-of-Sample Performance

The out-of-sample performance is tested on the options available on the deribit platform between 1 August 2018 and 15 August 2018. Options are selected according to the same criteria described in section 3. The final out-of-sample dataset consists of 29 call and 47 put option prices.

Results of the out-of-sample performance of the investigated models are illustrated in Table 2. At a first glance, one may notice that results linked to both absolute and relative performances change quite consistently. This is mainly due to the different structure of the out-of-sample dataset, in particular to the different maturities and market expectations.

**Table 2:** Out-of-sample performance of neural network and classical models.

	**TT**	**FDM**	**MC**	**NN**
**CALL**				
MAPE	0.0429	0.0429	0.0425	0.0283
MAE	26.64	26.65	26.77	17.93
MSE	1,016.11	1,016.28	1,026.79	441.94
**PUT**				
MAPE	0.0642	0.0643	0.0642	0.035
MAE	73.4	73.4	73.23	41.45
MSE	6,668.17	6,667.56	6,646.12	2,978.26

*The following notation is used: NN represents the neural network model, TT corresponds to the trinomial tree, FDM stands for finite difference method, and MC for the Monte Carlo simulation*.

**Figure 6:**
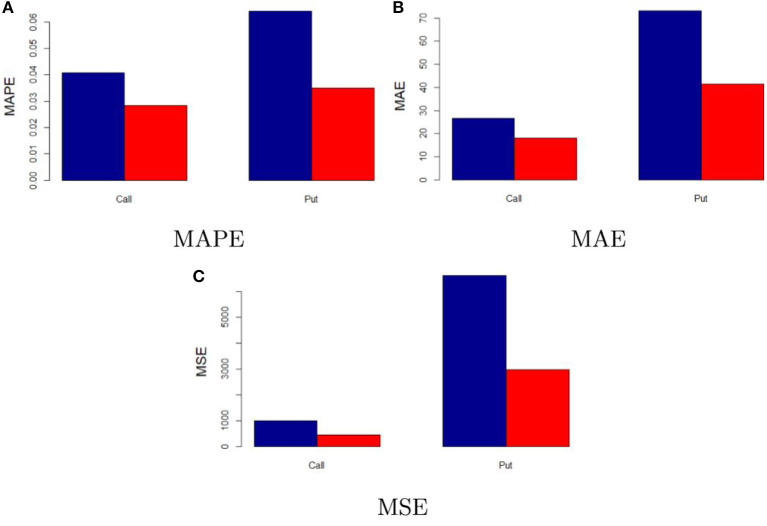
Out-of-sample performance of neural network and “best” classical model. The figure compares the out-of-sample performance of the neural network model (red) and “best” classical model (blue). **(A–C)** Illustrate the MAPE, MAE, and MSE, respectively.

As also depicted in Figure 6, it is clear that the neural network model proposed still outperforms the considered parametric methods. In addition, the difference in performance is even higher than the in-sample one. When comparing the performance of the neural network and the “best” classical model, the MAPE lowers by 33.41% in the call case and 45.48% in the put one, the MAE by 32.7% (call) and 43.4% (put) as well as the MSE by 55.23% (call) and 55.06% (put). This provides further support to the fact that the neural network is a feasible model to price Bitcoin options.

### 5.2. Cross-Validation

To further assess the robustness of our proposed model, the approach of repeated random sub-sampling for cross-validation purposes is adopted. In other words, the dataset is randomly split into training and validation set for 50 times and then the methodology and procedures described in this study are repeated. In this way one is able to determine whether the neural network performance achieved in the results section are stable, as well as to evaluate the model's relative performance after random sub-sampling with respect to the conventional option pricing methods.

**Figure 7:**
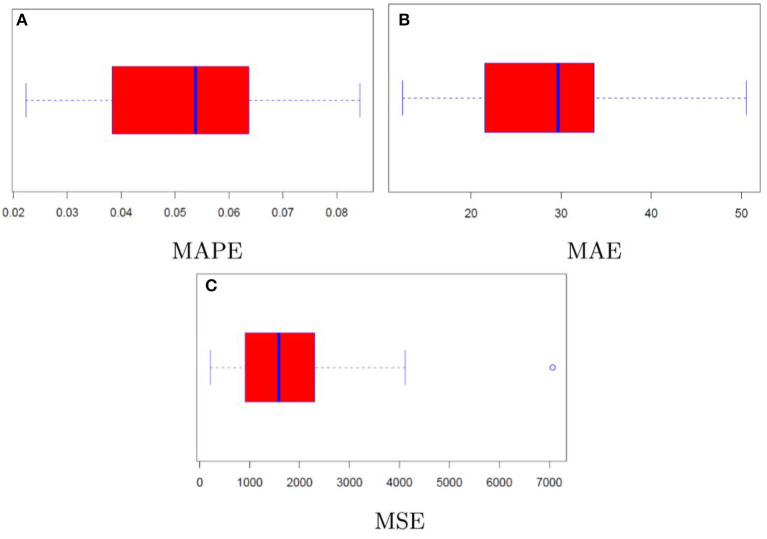
Model performance distribution (call). **(A–C)** Illustrate the MAPE, MAE, and MSE, respectively.

**Figure 8:**
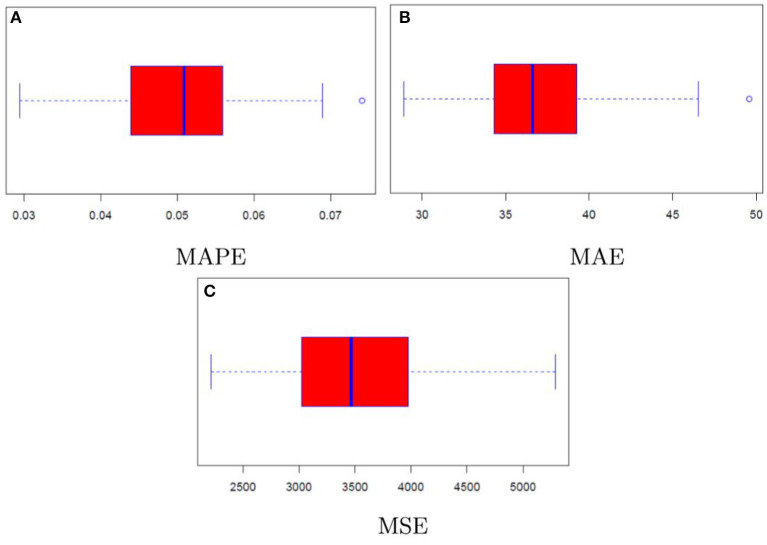
Model performance distribution (put). **(A–C)** Illustrate the MAPE, MAE, and MSE, respectively.

Results linked to the random sub-sampling procedure are illustrated through the boxplots contained in Figure 7 (call case) and Figure 8 (put case). Overall, outcomes are satisfactory provided that performance variability lies in ranges which are arguably not too wide. To illustrate, the interquartile ranges for MAPE and MAE are respectively <3% and below 10 USD in the call case, whereas in the put case they amount to roughly 1% and 5 USD.

Furthermore, comparing the distributions of the assessment criteria with the results in Table 1, it may be noticed that even in the context of resampling the neural network achieves again satisfactory results in terms of precision. Indeed, despite the MAPE results coming from the repeated random sub-sampling are partly worse than those of classical option pricing methods, the absolute assessment criteria still point to a substantial improvement when considering the neural network model rather than the conventional option pricing methods.

To conclude, there may be room for improvement in the modeling strategy, as well as this needs to be adapted to the specific case of interest. As an example, it can be argued that the neural network performances would benefit from increasing the number of observations and, specifically, by using high frequency data. In addition, as the market is highly volatile and the option market follows fast changing rules and patterns, different choices of the neural network specifications—different input layers, structure of the layers, activation functions, etc.,—may result more feasible in other contexts. Nevertheless, it may be claimed that the multilayer perceptron neural network model proposed is suitable for pricing options written on Bitcoin. Moreover, it may be argued that its application can be extended to the whole cryptocurrency framework, as well as to traditional markets.

## 6. Conclusion

This paper proposes an approach that relies on artificial neural network models for the purpose of Bitcoin option pricing. The methodology involves a first step in which options are priced according to some of the most widely employed parametric methodologies, i.e., tree models, Monte Carlo simulation, and finite difference method. The option prices obtained in this way are then used as input layers in a second step by the neural network, which is capable to refine the price predictions delivered by the parametric models in the first step. We believe that the proposed model can be extended, without loss of generality, to other cryptocurrency derivatives, as well as to traditional ones.

Empirical results show that the investigated conventional pricing methodologies yield to the conclusion that Bitcoin options are extensively overpriced. In contrast, by applying the proposed neural network model one is able to better represent the real market dynamics of Bitcoin option prices. Indeed, prediction errors consistently reduce when comparing the neural network pricing model to the classical parametric ones.

Further studies may benefit and improve prediction precision by using high frequency data as well as different model specifications. As an example, improvements could be achieved by the use of different models, such as stochastic volatility models, as input layers in the proposed neural network framework.

## Author Contributions

The author confirms being the sole contributor of this work and has approved it for publication.

### Conflict of Interest Statement

The author declares that the research was conducted in the absence of any commercial or financial relationships that could be construed as a potential conflict of interest.

## Footnotes

^1^“Altcoin” stands for “alternative coin.” The term is used to indicate all cryptocurrencies except for Bitcoin.

^2^Detailed information regarding the deribit BTC index can be found on www.deribit.com

^3^In particular, the following activation functions are tested: sigmoid, taylor, identity, tanh, softplus, gauss.
